# Comparison of statistical models for nested association mapping in rapeseed (*Brassica napus* L.) through computer simulations

**DOI:** 10.1186/s12870-016-0707-6

**Published:** 2016-01-25

**Authors:** Jinquan Li, Anja Bus, Viola Spamer, Benjamin Stich

**Affiliations:** Max Planck Institute for Plant Breeding Research, Carl-von-Linné-Weg 10, Köln, 50829 Germany; Syngenta Seeds GmbH, Zum Knipkenbach 20, Bad Salzuflen, 32107 Germany

**Keywords:** Statistical models, Nested association mapping (NAM), Rapeseed (*Brassica napus* L.), Double haploid NAM, Backcross NAM, Computer simulations

## Abstract

**Background:**

Rapeseed (*Brassica napus* L.) is an important oilseed crop throughout the world, serving as source for edible oil and renewable energy. Development of nested association mapping (NAM) population and methods is of importance for quantitative trait locus (QTL) mapping in rapeseed. The objectives of the research were to compare the power of QTL detection 1- *β*^∗^ (*β*^∗^ is the empirical type II error rate) (i) of two mating designs, double haploid (DH-NAM) and backcross (BC-NAM), (ii) of different statistical models, and (iii) for different genetic situations.

**Results:**

The computer simulations were based on the empirical data of a single nucleotide polymorphism (SNP) set of 790 SNPs from 30 sequenced conserved genes of 51 accessions of world-wide diverse *B. napus* germplasm. The results showed that a joint composite interval mapping (JCIM) model had significantly higher power of QTL detection than a single marker model. The DH-NAM mating design showed a slightly higher power of QTL detection than the BC-NAM mating design. The JCIM model considering QTL effects nested within subpopulations showed higher power of QTL detection than the JCIM model considering QTL effects across subpopulations, when examing a scenario in which there were interaction effects by a few QTLs interacting with a few background markers as well as a scenario in which there were interaction effects by many QTLs ($\geqslant 25$) each with more than 10 background markers and the proportion of total variance explained by the interactions was higher than 75 %.

**Conclusions:**

The results of our study support the optimal design as well as analysis of NAM populations, especially in rapeseed.

**Electronic supplementary material:**

The online version of this article (doi:10.1186/s12870-016-0707-6) contains supplementary material, which is available to authorized users.

## Background

Rapeseed (*Brassica napus* L.) is an important oilseed crop throughout the world, serving as source for edible oil and renewable energy. It is an amphidiploid (2n = 4x = 38, genome AACC) species which originated from a few interspecific hybridizations between *B. rapa* and *B. oleracea* [1]. This in turn led to a low genetic diversity in *B. napus*. The occurrence of two bottlenecks during rapeseed breeding, i.e. the selection for low erucic acid and low glucosinolate content further reduced the genetic diversity in modern elite varieties [2]. Low genetic diversity leads to genetic vulnerability [3] and reduces response to selection (cf. [4]). Therefore, it is desirable to introduce diverse germplasm into elite genetic material in rapeseed breeding programs and subsequently screen the material for performance traits.

The majority of phenotypic variation in natural populations and agricultural plants is due to quantitative traits [5]. An important step in genetics and breeding is to identify the genes contributing to the variation of such traits [6]. Linkage analysis and association mapping are two commonly used approaches to dissect the genetic basis of these quantitative traits [7]. In rapeseed, linkage mapping is a well-established approach and has been successfully applied for quantitative trait locus (QTL) mapping in bi-parental crosses (e.g. [8, 9]). Recently, association studies have become a promising approach in plant genetics to connect genetic polymorphisms with trait variations in diverse germplasm sets (e.g. [10, 11]). In rapeseed, several association studies have been carried out on the candidate-gene [12, 13] or on a genome-wide scale (e.g. [14–16]). Nested association mapping (NAM) has been suggested as a strategy to combine the high power of QTL detection from linkage analyses with the high mapping resolution of association mapping approaches [17]. In order to successfully use NAM, multi-parental mapping populations and statistical models are required.

Various mating designs were proposed for multi-parental mapping populations [17–19]. Among them, the NAM mating design has been successfully applied in maize [20]. To the best of our knowledge, no earlier study examined the possibility as well as the suitability of different mating designs for creating NAM populations in rapeseed. Moreover, as the current NAM mating design based on recombinant inbred lines (RIL-NAM) required several generations to develop RILs, new mating designs, which can shorten the time for generating NAM populations (for example, double haploid(DH) lines) or can increase the genetic background of common parent in the NAM progenies to fit for different types of germplasm resources, have not been examined yet.

Various statistical procedures have been applied for NAM. These QTL mapping methods included single marker models [21], interval mapping [22], composite interval mapping (CIM) [6], and recently proposed inclusive composite interval mapping (ICIM) [20, 23]. Such statistical models, however, should be examined for their usefulness in a specific species, especially under the situation of currently available high density linkage maps and large mapping data sets. Furthermore, in the context of NAM, the influence of QTL × genetic background interaction and varied sample sizes of subpopulations on the power of QTL detection has not yet been examined.

The objectives of this research were to compare the power of QTL detection 1- *β*^∗^ (i) of two mating designs, double haploid (DH-NAM) and backcross (BC-NAM), for the creation of NAM populations in rapeseed, (ii) of different statistical models, and (iii) for different genetic situations including various extents of QTL × genetic background interactions.

## Methods

### Parental genotypes

The computer simulations of this study were based on empirical data of 51 rapeseed genotypes of the Pre-Breeding Collection, which was constructed by Norddeutsche Pflanzenzucht Hans-Georg Lembke KG and German seed alliance, Germany from a world-wide diverse germplasm to catch maximum diversity. These genotypes can be divided into two panels. Panel 1 included the inbred entries PBY001(Pre-Breed Yield coding), PBY002, PBY003, PBY004, PBY007, PBY010, PBY011, PBY012, PBY013, PBY014, PBY015, PBY017, PBY018, PBY021, PBY022, PBY023, PBY024, PBY025, PBY026, PBY027, and PBY029. Panel 2 included PBY031, PBY032, PBY033, PBY034, PBY035, PBY036, PBY037, PBY038, PBY039, PBY040, PBY041, PBY043, PBY044, PBY045, PBY046, PBY047, PBY048, PBY049, PBY050, PBY051, PBY052, PBY053, PBY054, PBY055, PBY056, PBY057, PBY058, PBY059, PBY060 as well as the common parental line PBY061. The genotypes in panel 1 were genetically diverse but winter rapeseed inbreds adapted to German climate conditions, while the genotypes in panel 2 were exotic inbreds including winter, spring, and Swede rapeseed. The common parental line PBY061 was an elite winter rapeseed parent and wildly used as parent for commercial hybrid varieties.

### Computer simulations of parental genotypes

The single nucleotide polymorphisms (SNPs) were extracted from the sequences of the 30 conserved genes (Additional file 1) in all 51 genotypes. These genes were selected to get a population structure information of rapeseed germplasm resources that was influenced not too strongly by any recent selection effects. Based on the 30 conserved genes, the SNPs for the founders are homozygous. SNPs with a minor allele frequency of less than 5 % as well as the SNPs with 20 % of missing data were excluded from the study. Altogether 790 original SNPs were used for further analysis (Additional files 2 and 3). Genetic map distance information for these SNPs was lacking. Therefore, their genetic distance was calculated from the physical distance by a linear transformation with a rate of 0.674 Mb/cM according to [24]. The squared correlation of allele frequencies (*r*^2^) between SNP loci pairs was calculated to measure the level of linkage disequilibrium (LD) [25]. This measure was chosen as it can be interpreted as the proportion of variance which the allele frequency of the first marker explains of the allele frequency of the second marker [26]. A nonlinear regression of *r*^2^ versus the genetic map distance (cM) or physical distance (bp) was performed according to [27]. Furthermore, the modified Rogers distance (MRD) was calculated [28]. The distance was chosen because it is one of the most appropriate distance for codominant markers, such as SSR and SNP markers, and it has the Euclidean property which is important for principal coordinate analysis (PCoA). PCoA [29] based on MRD estimates between all pairs of inbred lines was performed for population structure.

Because of the limited number of SNPs available at the time when the study was performed, a total of 10,000 SNPs were simulated from the original SNPs. The simulated SNPs were evenly distributed across the genome. The number of SNPs on each chromosome was proportional to the length of the chromosome [24]. In order to create a set of SNPs that has similiar properties as the original set with respect to population structure and LD decay, the following strategy was applied. For each of the 10,000 SNPs, one SNP was randomly selected from the original SNP set and assigned to the simulated SNP locus. To break the strong LD between the original SNPs, random mating among the 51 parental inbreds was simulated to generate a random mating population with a total of 3000 individuals. Then 249 further generations of random mating were simulated among the random mating popolation with a constant population size of 3000 individuals. From each of these 3000 individuals, one DH line was simulated. A random sample of 51 individuals from the DH lines was drawn, and these simulated individuals were arbitrary assigned to each parent and considered in the following as the simulated parental inbreds. The analysis of the LD decay against genetic map distance and population structure within the simulated parental inbreds was performed with the aforementioned methods.

### Mating designs

The 51 simulated parental inbreds were used to examine two different mating designs using computer simulations. For the DH-NAM mating design, the 21 parental inbreds from panel 1 were crossed with the common parent PBY061, resulting in a total of 21 different F_1_ hybrids. A total of 100 DH individuals were generated from each F_1_. The final DH-NAM population consisted of a total of 2100 individuals. The mating design and the sample size were chosen because a population of such a size was under development in the framework of the Pre-BreedYied project supported by German Federal Ministry of Education and Research.

For the BC-NAM mating design, the 29 parental inbreds from panel 2 were crossed with the common parent PBY061, resulting in a total of 29 different F_1_ hybrids. Each hybrid was backcrossed once with the common parent PBY061 and generated 100 BC_1_ hybrids. The BC_1_ hybrids were selfed for two generations using the single seed descent (SSD) method to create a set of BC_1_S_2_ individuals. The final BC-NAM population consisted of a total of 2900 individuals. The BC_1_S_2_ generation was chosen to balance the percentage of homozygous lines in the population and the time for developing the population as well as because a population of such a size is under development in the frame of the Pre-BreedYied project.

To compare the power of QTL detection 1- *β*^∗^ of different mating designs with the same total population size, all 50 parental inbreds from both panels were applied to generate 50 DH-NAM subpopulations and 50 BC-NAM subpopulations using the two mating designs, respectively. In a scenario in which we compared the power of QTL detection 1- *β*^∗^ of the two mating designs and the NAM mating design based on recombinant inbred lines (RIL-NAM) [20], 50 RIL-NAM subpopulations were also simulated using all 50 parental inbreds, whereas the F_1_ hybrids were further selfed for 4 generations and created by SSD method. In a scenario in which we examined the influence of varied number of parental inbreds and mapping population sizes on the power of QTL detection 1- *β*^∗^, a subset of the size of 20 and 40 subpopulations with 100 individuals per subpopulation was randomly selected from all the subpopulations. A subset of the size of 40 subpopulations but only 50 individuals per subpopulation was also randomly selected. The power of QTL detection 1- *β*^∗^ of these mapping populations as well as all the 50 subpopulations was examined. In a scenario in which we examined the influence of unbalanced sample sizes of subpopulations on the power of QTL detection 1- *β*^∗^, a set of unbalanced sample sizes from a normal distribution with certain standard deviations (0, 5, 10, 20, 40) was applied to subpopulations while keeping the total number of individuals in the mapping population to 5000.

### Calculation of genotypic and phenotypic values

A total of 25 simulation runs were performed for each of the examined mating designs. For each run, three subsets of SNPs of the size *l* (*l*= 25, 50, 100) were randomly sampled without replacement from the genome and defined as QTL. The maximum genotypic effect per QTL q was drawn randomly without replacement from the geometric series 100(1-a) [1, a, a^2^, …, a ^*l*−1^] with a = 0.90 for 25 QTLs, a = 0.96 for 50 QTLs, or a = 0.99 for 100 QTLs [30]. To simplify, we treated rapeseed as a double diploid because its genome A and C have big difference, which is reasonable as current sequencing technology can effectively identify the SNPs from genome A or C. Therefore, for each SNP locus, only two alleles were assumed. The QTL effects for the two alleles were randomly given either by the maximum genotypic effect per QTL q or zero. The genotypic value of an individual was the sum of all of its QTL effects. Phenotypic values were generated by adding a realization from a normal distribution *N*(0, (1- *h*^2^) ${\sigma _{g}^{2}}/h^{2}$) to the genotypic values, where *h*^2^ denotes the heritability, and ${\sigma _{g}^{2}}$ is the genetic variance of all parental inbreds [19]. For our simulations *h*^2^= 0.5 and *h*^2^= 0.8 were assumed.

When examining the QTL × genetic background interactions, a total of 1, 5, 10, and 25 QTLs were randomly selected from the scenario of 50 QTLs. Each of these QTLs was assumed to have interaction effects with all the other non-QTL markers (1, 5, 10, 25). The proportion of total variance explained by the QTL × genetic background interaction was scaled to 5, 15, 25, 50, 75, and 95 % of the total genotypic variance.

### QTL mapping

Joint mapping, i.e. mapping using all populations at once, was used to identify QTLs. Four statistical models were used for QTL mapping. The first model was

$$y=b_{0}+a_{f}u_{f}+x_{q(f)b_{q(f)}}+e, $$

denoted as single marker model 1, where *y* was the vector of phenotypic values, *b*_0_ was the intercept, *u*_*f*_ was the effect of the cross of the founder *f* with the common parent, *a*_*f*_ was the incidence matrix relating each *u*_*f*_ to *y*, *x*_*q*(*f*)_ was a matrix of genotype of each individual in the subpopulation of the founder *f* at marker *q*, *b*_*q*(*f*)_ was the expected substitution effect of marker *q* in the subpopulation of the founder *f*, and *e* was the vector of residual variance. The second model was

$$y=b_{0}+a_{f}u_{f}+x_{q}b_{q}+e, $$

denoted as single marker model 2, where *y*, *b*_0_, *u*_*f*_, *a*_*f*_, and *e* were as described in single marker model 1, *x*_*q*_ was a vector of genotype of each individual at marker *q*, *b*_*q*_ was the expected substitution effect of marker *q*. The third model was

$${\small{\begin{aligned} y=b_{0}+a_{f}u_{f}+x_{q(f)}b_{q(f)}+\sum_{c \neq q} x_{c(f)}b_{c(f)}+e, \end{aligned}}} $$

denoted as joint composite interval mapping (JCIM) model 1, where *y*, *b*_0_, *u*_*f*_, *a*_*f*_, *x*_*q*(*f*)_, *b*_*q*(*f*)_, and *e* were as described in single marker model 1, *x*_*c*(*f*)_ was a matrix of genotype of each individual in the subpopulation of the founder *f* at cofactor *c* (cofactor *c*≠ marker *q*), *b*_*c*(*f*)_ was the expected substitution effect of cofactor *c* (cofactor *c*≠ marker *q*) in the subpopulation of the founder *f*. The fourth model was

$${\small{\begin{aligned} y=b_{0}+a_{f}u_{f}+x_{q}b_{q}+\sum_{c \neq q} x_{c}b_{c}+e, \end{aligned}}} $$

denoted as JCIM model 2, where *y*, *b*_0_, *u*_*f*_, *a*_*f*_, *x*_*q*_, *b*_*q*_, and *e* were as described in single marker model 2, *x*_*c*_ was a vector of genotype of each individual at cofactor *c* (cofactor *c*≠ marker *q*), *b*_*c*_ was the expected substitution effect of cofactor *c* (cofactor *c*≠ marker *q*).

Cofactor selection was performed using the LASSO function in the R package “lars” [31]. For doing so, a coefficient of variation for 10-fold cross-validation using the command cv.lars with default settings was computed and used for the LASSO function to select those independent variables (SNP markers) which have impact on the dependent variable (phenotype). In order to effectively screen cofactors in a large SNP set across the whole genome at lower computational cost, two methods were used for cofactor selection. We first cut each chromosome into 1.5 cM segments. This number was selected to balance the genomic interval density and the marker numbers for later calculation. Then, for the method 1, one marker was randomly selected from each segment for LASSO selection. Those markers having non-zero coefficients were kept as cofactors (denoted as cofactor 1). Based on the result of method 1, method 2 was applied to examine all the markers on the target segments which contained cofactors by method 1. All the markers on these target segments were selected and used for LASSO selection. Those markers having non-zero coefficients were kept as cofactors (denoted as cofactor 2). In brief, the method 1 detected whether there was one cofactor from each examined segment, while the method 2 detected whether there were more than one cofactor from those segments which contained cofactors by the method 1.

For QTL mapping, one by one of the 10,000 SNPs was used to fit the statistical models. For JCIM model 1 and 2, cofactor selection was performed prior to QTL mapping. During QTL mapping, when examined a certain SNP, the cofactors linked to the SNP within 5cM were excluded. The probability and effect for each examined SNP was obtained by analysis of variance (ANOVA) of the full model (with the examined SNP) against the residuals model (without the examined SNP).

### Power estimation method

The power of QTL detection 1−*β*^∗^ was calculated as follows, where *β*^∗^ is the empirical type II error rate and the symbol ^∗^ meant an empirical rate. As the SNPs that were considered as QTLs as well as the non-QTL markers were known in our computer simulations, we calculated the quantile of 0.5, 0.1, 0.01, 0.001, 0.0001, and 0.00001 of the probabilities for non-QTL markers (the nominal type I error rate *α*) and used the quantiles as the signicance threshold to identify a QTL, thus, a fixed empirical type I error rate *α*^∗^ of 0.5, 0.1, 0.01, 0.001, 0.0001, and 0.00001 was obtained. When a QTL had a probability less than the relavant quantiles, it was counted as a correctly identified QTL. The power of QTL detection 1−*β*^∗^ was calculated on the basis of these *α*^∗^ levels as proportion of correctly identified QTLs from the total number of QTLs [18]. This meant, the false positive rate was set to a known level (for example 5 %) when we calculated the power of QTL detection. The effects for the correctly identified QTLs (estimated effect) were taken to calculate the differnce of QTL effect, which was calculated by the following formular: $ D (\%) = \frac {\left | T - E \right | }{T} \times 100 $, where D was the difference of QTL effect, T was the true (simulated) QTL effect, and E was the estimated QTL effect by the models.

In a case where we compared the power of QTL detection 1−*β*^∗^ between the joint inclusive composite interval mapping (JICIM) model and the JCIM models, a same data set, i.e. 10 BC-NAM subpopulations with 50 QTLs, heritability *h*^2^=0.8 randomly selected from a total of 50 BC-NAM subpopulations, was used for both models. The analysis with JICIM model was followed by the manual of the software QTL IciMapping [32]. The missing phenotype was replaced by the mean of the trait as well as a step of 1 cM, a PIN value of 0.001 for stepwise regression selection, a logarithm of odds (LOD) threshold of 5.0, and the mapping method ICIM-ADD (JICIM) were selected. For JCIM analysis (model 1 and 2), only the cofactors selected by the Method 1 were used. All the non-polymorphic SNPs were excluded from the analysis. Similar to aforementioned method, the power of QTL detection 1−*β*^∗^ for the JICIM model was the proportion of correctly identified QTLs from the total number of QTLs. The empirical type I error rate *α*^∗^ was calculated by the proportion of false identified QTLs by JICIM model from the total number of non-QTL markers. The empirical type I error rate *α*^∗^ was further used to calculated the power of QTL detetion for the JCIM models according to the aforementioned method.

All the settings for the examined paraments were summarized in Table 1. If not stated differently, all analyses were performed with the statistical software R [33].

**Table 1:** Summary of the computer simulation settings. For details see ‘Methods’

Examined parameters	Setting values
Mating design	DH-NAM, BC-NAM, RIL-NAM
Statistical model	Single marker model 1 and 2, JCIM model 1 and 2
Cofactor selection	Method 1, Method 2
QTL number	1, 5, 25, 50, 100
Heritability	0.5, 0.8
Number of parens	20, 21, 29, 40, 50
Sample size per subpopulation	50, 100
Standard deviation for varied	
sample size per subpopulation	0, 5, 10, 20, 40
Explained percentage of variance	
by QTL × genetic background interaction	0 %, 5 %, 15 %, 25 %, 50 %, 75 %, 95 %
Number of QTL having	
QTL × genetic background interaction	1, 5, 10, 25
Number of background marker having	
QTL × genetic background interaction	1, 5, 10, 25

## Results

**Fig. 1:**
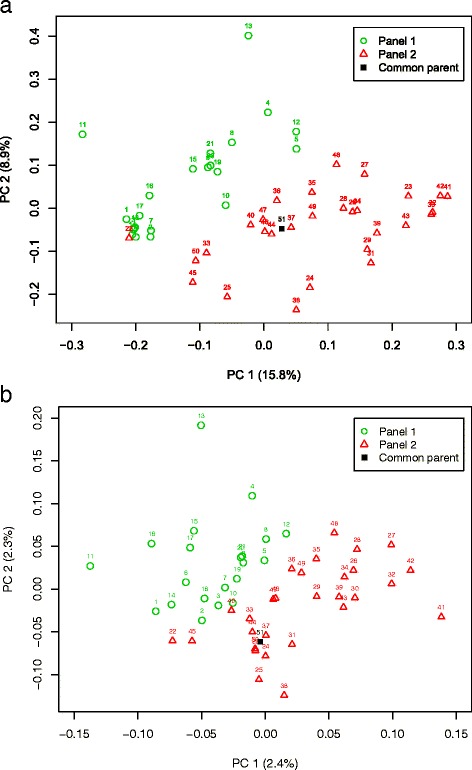
Principal coordinate analysis of the 51 parental inbreds based on (**a**) the original 790 SNPs from 30 conserved genes and (**b**) the simulated 10,000 SNPs. PC 1 and PC 2 refer to the first and second principal coordinates, respectively. The numbers in parentheses refer to the proportion of variance explained by the principal coordinates. Colors and symbols identify different sets of germplasm. The number 1–51 indicates the 51 of parental inbreds, i.e. PBY001-004, PBY007, PBY010-015, PBY017-018, PBY021-027, PBY029, PBY031-041, PBY043-061, respectively (see Methods). Number 51 is the common parental inbred used to simulate the nested association mapping populations

**Fig. 2:**
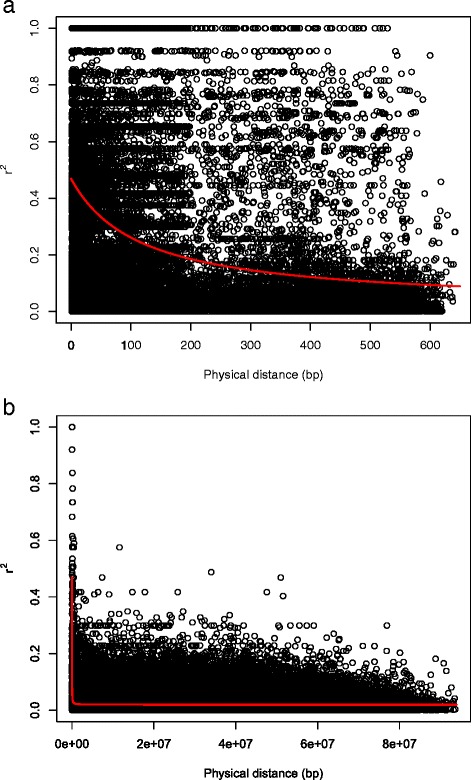
Nonlinear regression of the linkage disequilibrium measure *r* ^2^ against physical distance (bp) (**a**) based on the 790 original SNPs of the 51 parental inbreds and (**b**) based on 10,000 simulated SNPs of the simulated 51 parental inbreds. The red line is the nonlinear regression trend line of *r* ^2^ vs. physical distance

A total of 1605 SNPs were detected from the sequence of 30 conserved genes for the 51 parental inbreds, with a polymorphic rate of 11.19 %. Altogether 790 SNPs were retained after removing loci with a minor allele frequency of less than 5 % and used for the computer simulations. Based on these original SNPs, PCoA for the original parental inbreds revealed that the germplasm of panel 1 (adapted germplasm) and the germplasm of panel 2 (exotic germplasm) were located in two distinct clusters (Fig. 1a), and that the latter was more diverse than the former. Strong LD was observed between closely linked loci pairs (Fig. 2a). LD decayed to *r*^2^=0.1 within 545 bp, which corresponds approximately to a genetic map distance of 0.0008 cM. Based on the 10,000 simulated SNPs distributed across the genome (Additional files 4 and 5), the PCoA for the simulated parental inbreds revealed a pattern of population structure similar to that of the original parental inbreds (Fig. 1b). LD decayed to *r*^2^=0.1 within 0.08 cM (Fig. 2b).

**Fig. 3:**
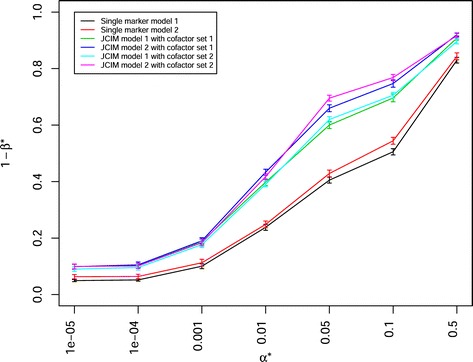
Power of QTL detection 1−*β* ^∗^ of four statistical models combined with two cofactor selection methods at different *α* ^∗^ levels in a scenario with 50 QTLs, heritability *h* ^2^= 0.8, and 40 backcross nested association mapping (BC-NAM) subpopulations which were randomly selected from a total of 50 BC-NAM subpopulations. JCIM represents joint composite interval mapping. Colors indicate different statistical models. Vertical lines at each point indicate the standard errors

For the scenario with 100 individuals in each of the 40 BC-NAM subpopulations, 50 QTLs, and *h*^2^= 0.8, the power of QTL detection 1−*β*^∗^ decreased with the empirical *α*^∗^ level decreasing from 0.5 to 0.00001 (Fig. 3, Table 2, Additional files 6 and 7). The statistical power of QTL detection 1- *β*^∗^ of single marker model 1 and 2, which did not include cofactors, was significantly lower than that of JCIM model 1 and 2, which included the selected cofactors. The statistical power of QTL detection 1- *β*^∗^ of the models using cofactor selection method 2 was slightly higher than that for the models using cofactor selection method 1. In case of a pure additively inherited trait, the statistical power of QTL detection 1- *β*^∗^ for the models considering the marker or cofactor effects nested within subpopulations (i.e. single marker model 1 and JCIM model 1) was lower than that for the models considering marker or cofactor effects across subpopulations (i.e. single marker model 2 and JCIM model 2). The power trends were similar for other examined scenarios, irrespective of mating designs, sample sizes, QTL numbers, and heritabilities. Moreover, for the difference between the estimated QTL effects by the statistical models and its relevant true (simulated) effects, the statistical model which had higher power of QTL detection (for example, JCIM model 2 with cofactor selection method 2) also had a lower difference of QTL effect than those models with lower power of QTL detection (Additional file 8).

**Table 2:** Summary of the nominal type I error rate *α* and power of QTL detection 1−*β* ^∗^ of four statistical models combined with two cofactor selection methods (C1, C2) at different *α* ^∗^ levels in a scenario with 50 QTLs, heritability *h* ^2^= 0.8, and 40 backcross nested association mapping (BC-NAM) subpopulations which were randomly selected from a total of 50 BC-NAM subpopulations, where *α* is the mean nominal type I error rate across the performed 25 simulation runs, *α* ^∗^ is the empirical type I error rate, S1 and S2 refer to single marker model 1 and 2, J1 and J2 refer to joint composite interval mapping model 1 and 2. For details see ‘Methods’

*α* ^∗^	S1			S2			J1C1			J2C1			J1C2			J2C2		
	*α*	1- *β* ^∗^		*α*	1- *β* ^∗^		*α*	1- *β* ^∗^		*α*	1- *β* ^∗^		*α*	1- *β* ^∗^		*α*	1- *β* ^∗^	
0.00001	9.71×10^−27^	0.049		4.51×10^−28^	0.063		1.09×10^−14^	0.099		4.00×10^−17^	0.099		6.40×10^−18^	0.090		1.41×10^−23^	0.100	
0.0001	9.68×10^−26^	0.052		5.69×10^−28^	0.064		5.01×10^−14^	0.102		3.99×10^−16^	0.105		5.35×10^−17^	0.096		1.41×10^−22^	0.101	
0.001	6.06×10^−19^	0.100		1.54×10^−21^	0.113		5.05×10^−11^	0.188		2.86×10^−13^	0.190		3.87×10^−12^	0.177		3.31×10^−16^	0.184	
0.01	6.41×10^−11^	0.238		2.50×10^−12^	0.248		5.17×10^−5^	0.398		1.68×10^−5^	0.433		3.23×10^−5^	0.391		5.25×10^−6^	0.417	
0.05	1.33×10^−6^	0.405		3.84×10^−7^	0.429		1.00×10^−2^	0.600		8.13×10^−3^	0.660		1.20×10^−2^	0.620		8.79×10^−3^	0.695	
0.1	8.12×10^−5^	0.506		3.53×10^−5^	0.544		4.22×10^−2^	0.696		3.86×10^−2^	0.747		4.94×10^−2^	0.706		4.51×10^−2^	0.768	
0.5	1.34×10^−1^	0.830		1.17×10^−1^	0.844		4.42×10^−1^	0.908		4.37×10^−1^	0.920		4.57×10^−1^	0.896		4.54×10^−1^	0.917	

**Fig. 4:**
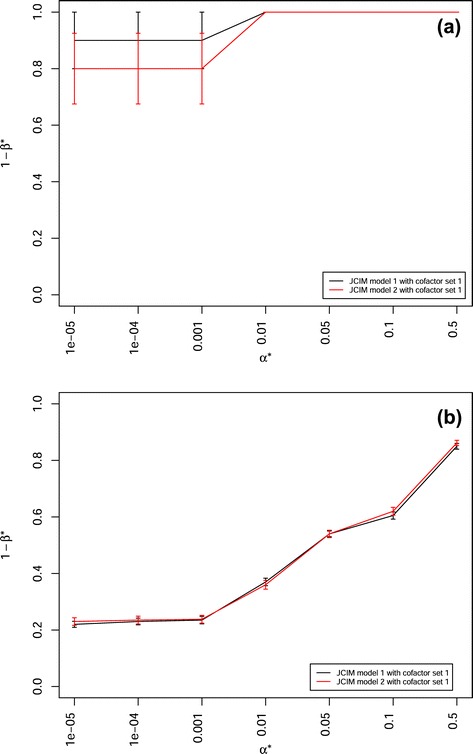
Power of QTL detection 1- *β* ^∗^ of joint composite interval mapping (JCIM) model 1 (black line) and 2 (red line) with cofactor selection method 1 at different *α* ^∗^ levels in a scenario with heritability *h* ^2^= 0.8, 50 backcross nested association mapping (BC-NAM) subpopulations and (**a**) 0.5 of explained ratio by QTL × genetic background interactions to the total genetic variance, where 1 QTL interacted with 5 background markers; (**b**) 0.75 of explained ratio by QTL × genetic background interactions to the total genetic variance, where each of 25 QTLs interacted with 10 background markers. Vertical lines at each point indicate the standard errors

However, the power of QTL detection 1- *β*^∗^ for JCIM model 1 was higher than that for JCIM model 2, when examing a scenario in which a few (1–5) QTLs had additive effects as well as QTL × genetic background interaction effects with a few background markers (≤5) and with a proportion of 50 % of the total variance explained by the interaction (Fig. 4a, Additional file 9), or a scenario in which there were interaction effects by many QTLs ($\geqslant 25$) with more than 10 background markers and the proportion of the total variance explained by the interactions was higher than 75 % (Fig. 4b, Additional file 10).

**Fig. 5:**
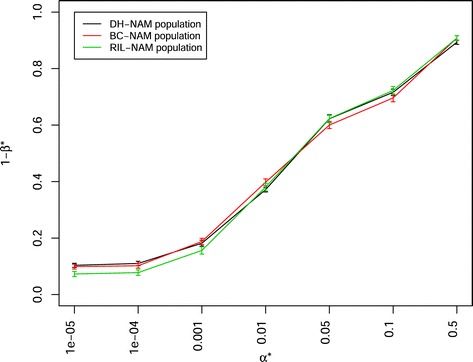
Power of QTL detection 1- *β* ^∗^ of joint composite interval mapping model 1 with cofactor selection method 1 for 40 double haploid nested association mapping (DH-NAM) vs. 40 backcross nested association mapping (BC-NAM) vs. 40 nest association mapping based on recombinant inbred lines (RIL-NAM) subpopulations at different *α* ^∗^ levels in a scenario with 50 QTLs and heritability *h* ^2^= 0.8. The 40 DH-NAM, BC-NAM or RIL-NAM subpopulations were randomly selected from a total of 50 relevant DH-NAM, BC-NAM, or RIL-NAM subpopulations. Colors indicate the DH-NAM, BC-NAM or RIL-NAM population. Vertical lines at each point indicate the standard errors

**Fig. 6:**
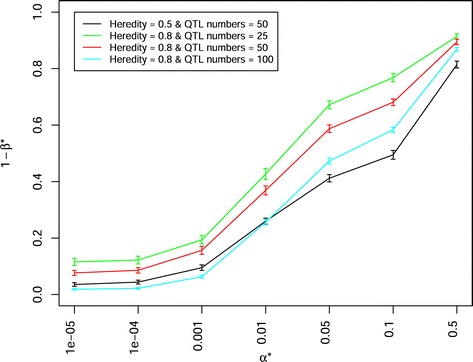
Power of QTL detection 1- *β* ^∗^ of joint composite interval mapping model 1 with cofactor selection method 1 for different numbers of QTLs (25, 50, 100) and different heritabilities (0.5, 0.8) at different *α* ^∗^ levels in a scenario of 29 backcross nested association mapping (BC-NAM) subpopulations. Colors indicate combinations of different number of QTLs and heritabilities. Vertical lines at each point indicate the standard errors

**Fig. 7:**
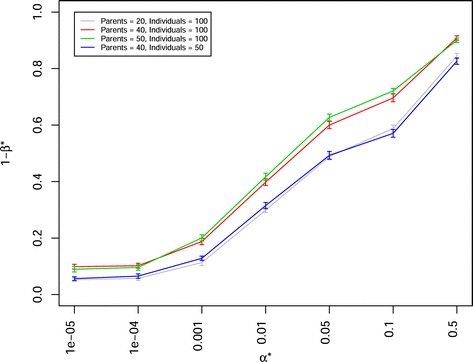
Power of QTL detection 1- *β* ^∗^ of joint composite interval mapping model 1 for different numbers of parental inbreds and backcross nested association mapping (BC-NAM) subpopulations (indicated by different colors) at different *α* ^∗^ levels in a scenario with 50 QTLs and heritability *h* ^2^= 0.8. Vertical lines at each point indicate the standard errors

**Fig. 8:**
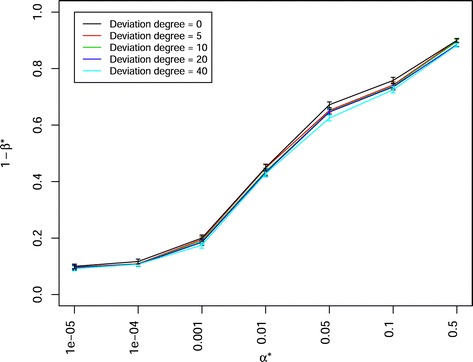
Power of QTL detection 1- *β* ^∗^ of joint composite interval mapping model 1 with cofactor selection method 1 for varied sample sizes of subpopulations with a standared deviation from 0 to 40 at different *α* ^∗^ levels in a scenario with 50 QTLs, heritability *h* ^2^=0.8, and 50 backcross nested association mapping (BC-NAM) subpopulations. Colors indicate different standard deviations for generating varied sample sizes of subpopulations. Vertical lines at each point indicate the standard errors

In a scenario in which the population sizes corresponded the sizes used in the Pre-BreedYield project to create 21 DH-NAM and 29 BC-NAM subpopulations (with 100 individuals for each subpopulation) were examined, the latter showed a significantly higher power of QTL detection 1- *β*^∗^ (e.g. 0.3785 at *α*^∗^=0.01) than the former (e.g. 0.2930 at *α*^∗^=0.01). When the number of involved parental inbreds and sample size was adjusted to the same value for both mating designs, DH-NAM and RIL-NAM mating designs showed a slightly (but not significantly) higher power of QTL detection 1- *β*^∗^ than BC-NAM mating design (Fig. 5, Additional files 6, 7, 11, 12, 13, 14). The trends for the power of QTL detection were similar, irrespective of QTL numbers, heritabilities, the numbers of parental inbreds, and sample sizes. The power of QTL detection 1- *β*^∗^ decreased significantly when the number of simulated QTLs increased from 25 to 100 (Fig. 6, Additional files 15, 16, 17, 18). Further, the power of QTL detection 1- *β*^∗^ significantly increased when the heritability was increased from 0.5 to 0.8. Similarly, the power of QTL detection 1- *β*^∗^ increased when the numbers of parental inbreds increased from 20 to 50 and the mapping population sizes increased from 2000 to 5000 (Fig. 7). With a constant total population size, the mapping population consisted of 40 subpopulations with 50 individuals per subpopulation showed a slightly (but not significantly) higher power of QTL detection 1- *β*^∗^ than the mapping population consisted of 20 subpopulations with 100 individuals per subpopulation (Fig. 7). The stronger the unbalancedness of the size of the individual subpopulation was, the lower was the power of QTL detection 1- *β*^∗^ (Fig. 8).

**Fig. 9:**
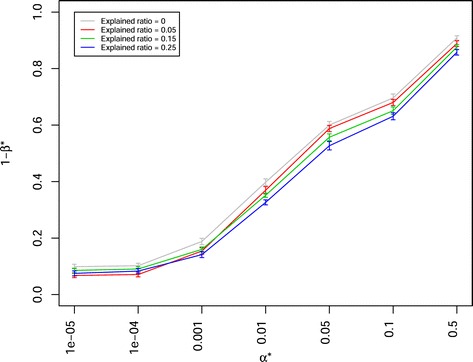
Power of QTL detection 1- *β* ^∗^ of joint composite interval mapping model 1 with cofactor selection method 1 for different explained ratio (0, 0.05, 0.15, 0.25) by QTL × genetic background interactions to the total genetic variance at different *α* ^∗^ levels in a scenario with 50 QTLs, heritability *h* ^2^= 0.8, and 40 backcross nested association mapping (BC-NAM) subpopulations. Colors indicate different explained ratio by QTL × genetic background interactions to the total genetic variance. Vertical lines at each point indicate the standard errors

The power of QTL detection 1- *β*^∗^ decreased when the proportion of the total genetic variance explained by QTL × genetic background interactions was increased from 0 to 0.25, irrespective of the mating designs, QTL numbers, heritabilies, and mapping population sizes (Fig. 9, Additional files 6, 7, 19, 20, 21).

A further comparison was performed for the power of QTL detection 1- *β*^∗^ between the JICIM model and JCIM model using the same mapping data (i.e. 10 BC-NAM subpopulations with 50 QTLs and heritability *h*^2^= 0.8) (Additional file 22). When the LOD value was set to 5.0 for JICIM, the empirical *α*^∗^ was close to 0.01 and the average power of QTL detection 1- *β*^∗^ was 0.052, which was much lower than those for JCIM model 1 ad 2 (0.219 and 0.266, respectively) at the same empirical *α*^∗^ levels.

## Discussions

### Simulation of parental inbreds

Rapeseed is one of the most important oilseed crops in the world. In order to efficiently select rapeseed varieties with improved yield and agronomic traits through marker or genomics-based selection, mapping of elite genes in diverse germplasm is required. This can be achieved by applying appropriate statistical methods that evaluate the association between genomic polymorphisms and phenotypic variation in different types of mapping populations [34].

Recently, the nested association mapping strategy was suggested to combine the high power of QTL detection from linkage analyses with the high mapping resolution of association analysis [17]. The strategy is based on RIL populations derived from crosses between a set of parental inbreds and one common parent from a diverse germplasm set. However, the evaluation of the NAM strategy or other NAM-like strategies requires developing, genotyping, and phenotyping large RIL populations, which in turn requires large financial resources (cf. [20]). Therefore, computer simulations are mandatory for examining the properties and evaluating the performance of the different described statistical models and methods.

We observed a total of 1605 SNPs from the sequences of 30 conserved genes for the parental inbreds, with a polymorphic rate of 11.19 %, which means about 1 SNPs per 9.1 bp. The polymorphic rate found in our study was considerably higher than that reported in prevous stuides [35, 36]. The difference might be explained by the large number of inbreds (51 parental inbreds) and the highly diverse germplasm (including exotic and adapted germplasm) that was used for SNP detection in our study.

To check LD decay, we made a nonlinear regression of *r*^2^ versus the genetic map distance (cM) or physical distance (bp) according to [27] and calculated the distance when *r*^2^=0.1. We observed that LD decayed on average within 545 bp to *r*^2^=0.1. This number of bp corresponds roughly to 0.0008 cM. The LD decay in our study was much faster than in the studies of [37] and [38], where [37] found that the expected *r*^2^ declined to the significance threshold (95th quantile of *r*^2^ for unlinked loci) within about 1 cM in a diverse germplasm set, and [38] found high levels of LD extending over about 2 cM in a set of 85 winter oilseed rape types. The difference might be explained by the following reasons. Firstly, different thresholds were applied to measure LD decay. Secondly, in our study LD decay within conserved genes was examined, whereas the previous researches studied genome-wide LD decay inferred from molecular markers. Thirdly, all studies were done on different sets of germplasm.

Based on the global LD decay (within 1cM) in a large and diverse rapeseed population, assuming a genome size of at least 2,000 cM, and aiming at a coverage of at least 1 marker per cM, the research of [37] suggested that considerably more than 2,000 markers would be required for genome-wide association studies. At the beginning of our project, we only had the sequences of the 30 conservative genes. With the information from these sequences, we could well examine the population structure of the founder lines and know the allele frequencies among the founder lines as well. This information provided the basis for our computer simulations. Though 30 conserved genes do not reflect the true genomic situation, however, it reflects better the genomic situation than ignoring this information. Therefore, a total of 10 000 SNPs were simulated based on the original SNPs from the conserved genes. Such a simulated SNP set should have as similar properties as possible as the original set with respect to population structure and LD decay. We observed that our simulated parental inbreds maintained allele frequencies, and therewith also population structure, similar to the original parental inbreds (Fig. 1b). The LD decay in the simulated parental inbreds is about 100 times slower than that in the original parental inbreds (Fig. 2b). Though we could make more generations of random mating to get the same LD decay as the original SNP set, this would require considerable computational time and resources. This has the potential to lead to a higher power of QTL detection when using the simulated parent inbreds as the parents of NAM population rather than the original parental inbreds for all examined scenarios. However, the ranking for the power of QTL detection 1- *β*^∗^ for the examined scenarios is expected not to change. Therefore we think that the simulation of parental inbreds is a legitimate approach for the questions to be examined in our study.

### Comparison of power of QTL detection 1- *β*^∗^ of the examined statistical models for NAM

The single marker model was frequently used in the early association mapping research. This model was used in our study as a reference. We further introduced the JCIM model in our study, where, similar to CIM, markers were chosen as cofactors to control the genetic variation of the genetic background. Considering the structure of the NAM populations, we included a subpopulation effect in all examined models.

As (1) the ranking of the examined statistical models with respect to the power of QTL detection 1- *β*^∗^ was the same in all examined scenarios except the scenarios with QTL × genetic background interactions, and (2) the BC-NAM mating design is important for plant breeder to make use of the exotic germplasm resouces, we discuss in the context of the comparison of statistical methods for QTL detection only the results of the scenario with 40 BC-NAM subpopulations (100 individuals per subpopulation), 50 QTLs, and heritability *h*^2^=0.8.

We observed that the power of QTL detection 1- *β*^∗^ for the JCIM model 1 and 2, which used cofactors, was significantly higher than that of single marker model 1 and 2, which did not use cofactors (Fig. 3). However, the former statistical models required much more computational effort to screen cofactors than the latter. The higher power of QTL detection 1- *β*^∗^ for the JCIM models than the single marker models can be explained by the fact that cofactors not only corrected for population stratification, but also for the genetic variation of other possibly linked or unlinked QTL which led to an increasing QTL detection power and better estimation of QTL effects [6]. Moreover, we observed a high power of QTL detection 1- *β*^∗^ using cofactor 2 (selected by the method 2) and smaller difference of QTL effects between the estimated and true QTL effects than those using cofactor 1 (selected by the method 1). This might be explained by the difference of the two cofactor selection methods. The method 1 detected only one marker from each examined segment to be cofactor or not, whereas the method 2 used all markers for cofactor selection from the segments containing cofactors previous identified by the method 1. Therefore, more appropriate markers were used as cofactors from each examined segment for the latter than the former. However, the latter required much more computational effort to screen cofactors than the former. Our results indicate that the proposed cofactor selection method, which was executed by LASSO function [39], was highly efficient with regard to computation time even when dealing with a large number of variables. In the following we only discuss the results of the JCIM models with cofactor selection method 1.

We observed that the power of QTL detection 1- *β*^∗^ for the JCIM model 1 was significantly lower than that for the JCIM model 2 when the QTLs had only additive effects (Fig. 3). This was also true when the QTLs had additive effects plus interaction effects with a low or medium proportion of the total genetic variance explained by many QTLs × genetic background interactions. The former model considered marker or cofactor effects nested within subpopulations, whereas the latter model considered marker effects across subpopulations. This might be because more parameters need to be estimated for the former than the latter model, which in turn reduces the power of QTL detection.

However, the power of QTL detection 1- *β*^∗^ for JCIM model 1 was higher than that for JCIM model 2 when examing a scenario in which there were interaction effects by a few (1–5) QTLs interacting with a few background markers (≤5) (Fig. 4a) as well as a scenario in which there were interaction effects by many QTLs ($\geqslant 25$) with more than 10 background markers and the proportion of the total variance explained by the interactions was higher than 75 % (Fig. 4b).

In a NAM population, not all families have segregating alleles at a given SNP locus, which might result in different degrees of freedom of tested models and different levels of probabilities for examined markers. However, this will not affect the ranking of the examined statistical models with respect to the power of QTL detection 1- *β*^∗^ in our study. The reasons are: (1) in our simulations, the power to detect QTL at certain empirical type I error rates *α*^∗^ were compared. The power of QTL detection 1- *β*^∗^ mainly relies on the ranking of probabilities for non-QTL markers, which will not be affected by the degree of freedom of the tested models (for details see Methods); (2) furthermore, when we compared the QTL detection power to detect QTL for each scenario, we were based on the same segregating families, which had the same segregation alleles at a given SNP locus.

In empirical studies where the extent of QTLs × genetic background interactions is unknown, the JCIM model 2 is suggested to be applied for a primary scan, as the model in most cases showed a higher power of QTL detection than JCIM model 1. Based on the results from the primary scan, a secondary scan is suggested to be applied using the JCIM model 1 in a scenario in which there were interaction effects by a few (1–5) QTLs interacting with a few background markers (≤5) as well as a scenario in which there were complicated interaction effects such as many QTLs ($\geqslant 25$) with several background markers.

Moreover, we observed that our proposed JCIM models showed higher power of QTL detection than the existing JICIM model (Additional file 22). The reason might be due to that (1) our proposed cofactor selection methods could effectively select cofactors to control the variation of genetic bakcground during QTL mapping, and (2) our proposed models could effectively control the impact of population structure. However, as the examined parameters and mapping procedures are different for these models, a comprehensive comparison among the existing statistical models for NAM analysis should be performed in future.

### Influence of mating designs on power of QTL detection

In the Pre-BreedYield project, two different sets of germplasm, namely adapted and exotic germplasm, were used. Accordingly, a DH-NAM mating design was applied to the adapted germplasm because it required little time to create fully homozygous genotypes via DH development and thus make use of the elite germplasm resources. However, for the exotic germplasm, due to the likely reasons of low compatibility, hybrid sterility, linkage drag, and inferior performance of hybrids, it might require more generations of backcrossing and selfing to overcome these obstacles. In such cases, the BC-NAM mating design might be appropriate when using exotic germplasm as introgression donor parents. Therefore, the power of QTL detection 1- *β*^∗^ for the two mating designs were compared in the study.

In a scenario of 40 DH-NAM vs. 40 BC-NAM subpopulations with 50 QTLs, heritability *h*^2^= 0.8, and 100 individuals per subpopulation, we observed that the DH-NAM mating design showed a slightly, but not significantly, higher power of QTL detection 1- *β*^∗^ than the BC-NAM mating design, irrespective of QTL numbers and heritabilities examined (Fig. 5). This difference in power estimates between the two mating designs might be due to that the average allele frequencies for the DH-NAM population were close to 0.5, whereas for the BC-NAM design the common parental inbred had a higher allele frequency than the donor parental inbreds. This in turn leads to a higher power of QTL detection for the DH-NAM mating design than for the BC-NAM mating design. The explanation could be supported by the findings of [19], who observed that the differences in allele frequencies for different crossing schemes contributed to the difference in power estimates.

### Influence of different genetic parameters on power of QTL detection

In this study we examined the influence of (i) the genetic architecture of the examined traits, (ii) the mapping population size and the number of parental inbreds, and (iii) unbalancedness of the size of the subpopulations on the power of QTL detection 1- *β*^∗^.

*Genetic architecture of the trait:* We observed a higher power of QTL detection 1- *β*^∗^ for the traits assessed with a high heritability than for the traits assessed with a low heritability (Fig. 6). Similiar trends were observed for the traits controlled by a low number of QTLs than for the traits controlled by a high number of QTLs. The reason is that in the former case each QTL explained a higher proportion of the phenotypic variance than in the latter. For the traits influenced by QTLs with both additive effects and QTL × genetic background interaction, the power of QTL detection was significantly lower than for those influenced by QTLs with purely additive effects (Fig. 9). Moreover, the higher the proportion of the total variance explained by QTL × genetic background interaction was, the lower was the power of QTL detection 1- *β*^∗^. Our observation was similar to the research of [40] and could be explained by the fact that a high proportion of the total variance by QTL × genetic background interaction reduced the proportion of the phenotypic variance explained by each QTL, and thereby reduced the power of QTL detection [18].

*Mapping population size and the number of parental inbreds:* Across all examined scenarios, a higher power of QTL detection 1- *β*^∗^ was observed for the mapping populations with a higher number of individuals and parental inbreds (Fig. 7). This observation was in accordance with the results of [18] and could be explained by the fact that in this case allele effects are estimated more precisely, and that a higher number of parental inbreds increased the number of polymorphic QTL [18]. Furthermore, with a constant total population size, the mapping population consisted of 40 subpopulations with 50 individuals per subpopulation showed a slightly (but not significantly) higher power of QTL detection 1- *β*^∗^ than the mapping population consisted of 20 subpopulations with 100 individuals per subpopulation (Fig. 7). The reason for this might be that a higher number of subpopulations leads to a higher number of parental inbreds and polymorphic QTL in the total mapping population for the former than the latter, and this in turn inceases the power of QTL detection 1- *β*^∗^.

*Unbalancedness of the size of the subpopulations:* We further examined the influcence of unbalancedness of the size of the subpopulations on the power of QTL detection 1- *β*^∗^. Our results suggested that a mapping population with an unbalanced size of the subpopulations had a significantly lower power of QTL detection 1- *β*^∗^ than that with a balanced size of the subpopulations, although the total size of the mapping population was the same (Fig. 8). The reason for this might be that an unbalanced size of subpopulations leads to an unbalanced frequency of the alleles of the individual parental inbred in the total mapping population, and this in turn has the potential to reduce the power of QTL detection 1- *β*^∗^.

As no earlier studies reported results from nested association mapping in rapeseed, our research is indispensable to draw conclusions about the prospects of nested association mapping in rapeseed. The results of our study support the optimal design as well as analysis of NAM populations, especially in rapeseed. As nested association mapping can efficiently combine the advantages of linkage mapping and association mapping, the developed statistical models for NAM in this study is of importance for detecting novel QTLs and preparing marker assisted selection programs in rapeseed.

## Conclusions

Our research showed that a joint composite interval mapping (JCIM) model had significantly higher power of QTL detection than a single marker model. DH-NAM mating design showed a slightly higher power of QTL detection than the BC-NAM mating design. The JCIM model considering QTL effects nested within subpopulations showed higher power of QTL detection than the JCIM model considering QTL effects across subpopulations, when examing a scenario in which there were interaction effects by a few QTLs interacting with a few background markers as well as a scenario in which there were interaction effects by many QTLs ($\geqslant 25$) each with more than 10 background markers and the proportion of total variance explained by the interactions was higher than 75 %, vise versa. The results of our study support the optimal design as well as analysis of NAM populations, especially in rapeseed.

## Availability of supporting data

The data sets supporting the results of this article are included within the article and its additional files (Additional files 1, 22, 8: see supplementary materials; Additional files 2, 3, 4, 5, 6, 7, 9, 10, 11, 12, 13, 14, 15, 16, 17, 18, 19, 20, 21, i.e. Table S3 - Table S21, deposited in the public repository Figshare with DOI: https://dx.doi.org/10.6084/m9.figshare.2009268).

## Additional files

Additional file 1
**Table S1.** The information of the 30 conserved genes and their annotations in *Arabidopsis thaliana* genome. Description: For those having no gene annotation in *A. thaliana* genome, the start and end positions in *A. thaliana* genome were given. (PDF 14.9 Kb)

Additional file 2
**Table S20.** Genotypes for the 51 parental inbreds at the original SNPs. (TXT 186 kb)

Additional file 3
**Table S21.** A map file for the original SNPs. (TXT 31 kb)

Additional file 4
**Table S3.** Genotypes for the simulated 51 parental inbreds at the simulated 10 K SNPs. (XLSX 31 kb)

Additional file 5
**Table S4.** Marker name and its position at chromosome for the simulated 10 K SNPs. (XLSX 3133 kb)

Additional file 6
**Table S5.** Genotypes for the 50 BC-NAM subpopulations, where 0 and 2 denote the homozygous gentoype of parents, respectively, while 1 denotes the heterozygous genotype. (TXT 88,883 kb)

Additional file 7
**Table S8.** Phenotypes for the 50 BC-NAM subpopulations in a scenario where QTLs = 50, *h* ^2^=0.8 with 25 replications. (TXT 2088 kb)

Additional file 8
**Figure S1.** Percentage of difference between estimated and true (simulated) QTL effects for joint composite interval mapping (JCIM) model 1 and 2 with cofactor selection method 1 and 2 in a scenario with 50 QTLs, heritability *h* ^2^= 0.8, and 40 backcross nested association mapping (BC-NAM) subpopulations. Description: Colors indicate different statistical models and different cofactor sets. Vertical lines at each point indicate the standard errors. (PDF 8.01 Kb)

Additional file 9
**Table S9.** Phenotypes for the 50 BC-NAM subpopulations in a scenario where QTLs = 1 which interacted with 5 background markers, *h* ^2^=0.8, 0.5 of explained ratio by QTL × genetic background interactions to the total genetic variance with 25 replications. (TXT 2078 kb)

Additional file 10
**Table S10.** Phenotypes for the 50 BC-NAM subpopulations in a scenario where QTLs = 25, *h* ^2^=0.8, 0.75 of explained ratio by QTL × genetic background interactions to the total genetic variance, each of 25 QTLs interacted with 10 background markers with 25 replications. (TXT 2078 kb)

Additional file 11
**Table S6.** Genotypes for the 50 DH-NAM subpopulations, where 0 and 2 denote the homozygous gentoypes of parents, respectively. (TXT 88,883 kb)

Additional file 12
**Table S7.** Genotypes for the 50 RIL-NAM subpopulations, where 0 and 2 denote the homozygous gentoype of parents, respectively, while 1 denotes the heterozygous genotype. (TXT 88,883 kb)

Additional file 13
**Table S14.** Phenotypes for the 50 DH-NAM subpopulations in a scenario where QTLs = 50, *h* ^2^=0.8 with 25 replications. (TXT 2088 kb)

Additional file 14
**Table S15.** Phenotypes for the 50 RIL-NAM subpopulations in a scenario where QTLs = 50, *h* ^2^=0.8 with 25 replications. (TXT 2078 kb)

Additional file 15
**Table S16.** Phenotypes for the 29 BC-NAM subpopulations in a scenario where QTLs = 50, *h* ^2^=0.5 with 25 replications. (TXT 1208 kb)

Additional file 16
**Table S17.** Phenotypes for the 29 BC-NAM subpopulations in a scenario where QTLs = 25, *h* ^2^=0.8 with 25 replications. (TXT 1208 kb)

Additional file 17
**Table S18.** Phenotypes for the 29 BC-NAM subpopulations in a scenario where QTLs = 50, *h* ^2^=0.5 with 25 replications. (TXT 1208 kb)

Additional file 18
**Table S19.** Phenotypes for the 29 BC-NAM subpopulations in a scenario where QTLs = 100, *h* ^2^=0.5 with 25 replications. (TXT 1208 kb)

Additional file 19
**Table S11.** Phenotypes for the 50 BC-NAM subpopulations in a scenario where QTLs = 50, *h* ^2^=0.8, 0.05 of explained ratio by QTL × genetic background interactions to the total genetic variance with 25 replications. (TXT 2078 kb)

Additional file 20
**Table S12.** Phenotypes for the 50 BC-NAM subpopulations in a scenario where QTLs = 50, *h* ^2^=0.8, 0.15 of explained ratio by QTL × genetic background interactions to the total genetic variance with 25 replications. (TXT 2088 kb)

Additional file 21
**Table S113.** Phenotypes for the 50 BC-NAM subpopulations in a scenario where QTLs = 50, *h* ^2^=0.8, 0.25 of explained ratio by QTL × genetic background interactions to the total genetic variance with 25 replications. (TXT 2088 kb)

Additional file 22
**Table S2.** Comparisions of the power of QTL detection 1- *β* ^∗^ among the joint inclusive composite interval mapping (JICIM) model, joint compositve interval mapping (JCIM) model 1 and 2. Description: The comparisons were performed in a scenario with 50 QTLs, heritability *h* ^2^= 0.8, cofactors selected by the Method 1, and 10 backcross nested association mapping (BC-NAM) subpopulations which were randomly selected from a total of 50 BC-NAM subpopulations. The empirical type I error *α* ^∗^ was calculated based the mapping results from JICIM model and the segregating markers and QTLs within the mapping population. For details see Methods. (PDF 16.1 kb)

## Abbreviations

ANOVA: Analysis of variance
BC-NAM: Backcross nested association mapping
CIM: Composite interval mapping
cM: centi Morgen
DH: Double haploid
DH-NAM: Double haploid nested association mapping
ICIM: Inclusive composite interval mapping
JCIM: Joint composite interval mapping
JICIM: Joint inclusive composite interval mapping
LASSO: Least absolute shrinkage and selection operator
LD: Linkage disequilibrium
LOD: Logarithm of odds
MRD: Modified Rogers distance
NAM: Nested association mapping
PBY: Pre-breed yield
PCoA: Principal coordinate analysis, QTL: Quantitative trait locus
RIL: Recombinant inbred line
RIL-NAM: Recombinant inbred lines nested association mapping
SSD: Single seed descent
SNP: Single nucleotide polymorphism
