# Supplementary material for: Comparison of statistical models for nested association mapping in rapeseed (Brassica napus L.) through computer simulations
Source: BMC Plant Biol. 2016 Jan 25;16:26. doi: 10.1186/s12870-016-0707-6 (PMC4727311; doi:10.1186/s12870-016-0707-6)
Supplement: Additional file 1 — Table S1. The information of the 30 conserved genes and their annotations in Arabidopsis thaliana genome. Description: For those having no gene annotation in A. thaliana genome, the start and end positions in A. thaliana genome were given. (PDF 14.9 Kb) [file 12870_2016_707_MOESM1_ESM.pdf]

**Table S1** The information of the 30 conserved genes and their annotations in *Arabidopsis thaliana* genome, where for those having no gene annotation in *A. thaliana* genome, the start and end positions in *A. thaliana* genome were given.

| Primer | Chromosome of *Brassica napus* | Type | Gene in *A. thaliana* | Chromosome of *A. thaliana* | Subject Start | Subject End |
|---|---|---|---|---|---|---|
| HiNK22853 | A01 | COS | AT4G14342 | Chr4 | 8253550 | 8255508 |
| HiNK22859 | A02 | STS | AT5G17920 | Chr5 | 5835038 | 5939487 |
| HiNK22857 | A02 | Random | AT1G75810 | Chr1 | 28461664 | 28462366 |
| HiNK22860 | A02 | COS | AT5G22360 | Chr5 | 7404004 | 7405765 |
| HiNK22861 | A03 | STS | AT4G00585 | Chr4 | 250955 | 252598 |
| HiNK22865 | A04 | STS | AT2G27290 | Chr2 | 11678400 | 11679797 |
| HiNK22866 | A04 | STS | AT3G63140 | Chr3 | 23326867 | 23328709 |
| HiNK22869 | A05 | Random | - | Chr5 | 10362900 | 10362807 |
| HiNK22872 | A06 | STS | AT3G04920 | Chr3 | 1360882 | 1362295 |
| HiNK22874 | A06 | STS | AT3G49100 | Chr3 | 18196839 | 18198457 |
| HiNK22877 | A07 | STS | AT4G24780 | Chr4 | 12770331 | 12772479 |
| HiNK22875 | A07 | Random | AT2G02680 | Chr2 | 745775 | 748573 |
| HiNK22882 | A08 | COS | AT1G42970 | Chr1 | 16127381 | 16129843 |
| HiNK22883 | A09 | COS | AT1G30630 | Chr1 | 10858298 | 10860261 |
| HiNK22888 | A10 | Random | AT1G02260 | Chr1 | 440441 | 442877 |
| HiNK22786 | C00 | STS | AT3G59530 | Chr3 | 21993253 | 21995308 |
| HiNK22787 | C01 | Random | - | Chr4 | 17735254 | 17735412 |
| HiNK22788 | C01 | Random | AT3G17465 | Chr3 | 5977817 | 5979652 |
| HiNK22793 | C02 | STS | AT5G46030 | Chr5 | 18670117 | 18671373 |
| HiNK22797 | C03 | STS | AT3G44680 | Chr3 | 16226556 | 16229954 |
| HiNK22799 | C04 | COS | AT2G27450 | Chr2 | 11737432 | 11739690 |
| HiNK22800 | C04 | Random | - | Chr1 | 16105441 | 16105495 |
| HiNK22804 | C05 | STS | AT1G70580 | Chr1 | 26612632 | 26616069 |
| HiNK22803 | C05 | COS | AT3G06610 | Chr3 | 2060737 | 2062067 |
| HiNK22805 | C06 | Random | - | Chr3 | 14864155 | 14863837 |
| HiNK22806 | C06 | Random | - | Chr1 | 26213761 | 26213585 |
| HiNK22807 | C06 | Random | - | Chr2 | 4631826 | 4630468 |
| HiNK22814 | C08 | Random | AT1G17890 | Chr1 | 6154268 | 6155752 |
| HiNK22816 | C09 | Random | - | Chr5 | 15962649 | 15962562 |
| HiNK22817 | C09 | Random | AT5G20840 | Chr5 | 7061103 | 7069219 |
