# Supplementary figures and images for: Comparison of statistical models for nested association mapping in rapeseed (Brassica napus L.) through computer simulations
Source: BMC Plant Biol. 2016 Jan 25;16:26. doi: 10.1186/s12870-016-0707-6 (PMC4727311; doi:10.1186/s12870-016-0707-6)

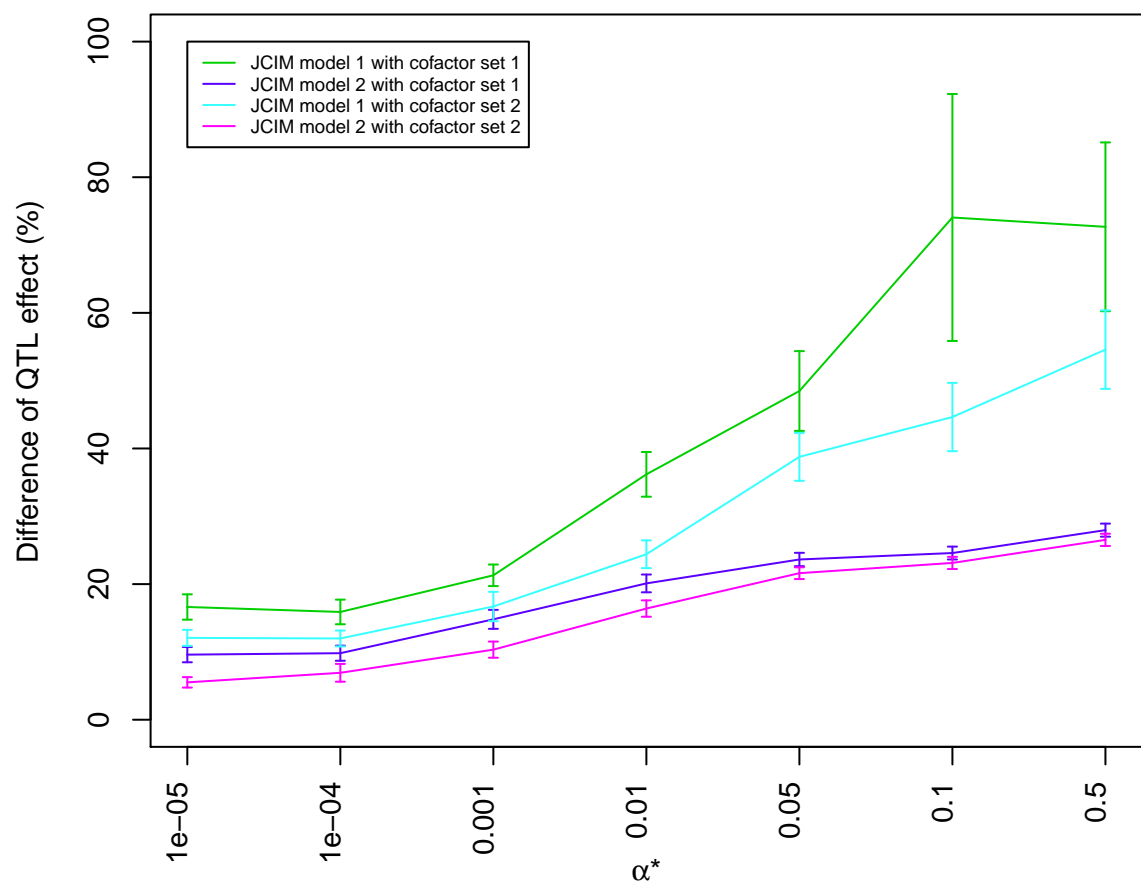

Supplement: Additional file 8 — Figure S1. Percentage of difference between estimated and true (simulated) QTL effects for joint composite interval mapping (JCIM) model 1 and 2 with cofactor selection method 1 and 2 in a scenario with 50 QTLs, heritability h 2= 0.8, and 40 backcross nested association mapping (BC-NAM) subpopulations. Description: Colors indicate different statistical models and different cofactor sets. Vertical lines at each point indicate the standard errors. (PDF 8.01 Kb) [file 12870_2016_707_MOESM8_ESM.pdf]
