# Supplementary material for: Comparison of statistical models for nested association mapping in rapeseed (Brassica napus L.) through computer simulations
Source: BMC Plant Biol. 2016 Jan 25;16:26. doi: 10.1186/s12870-016-0707-6 (PMC4727311; doi:10.1186/s12870-016-0707-6)
Supplement: Additional file 22 — Table S2. Comparisions of the power of QTL detection 1- β ∗ among the joint inclusive composite interval mapping (JICIM) model, joint compositve interval mapping (JCIM) model 1 and 2. Description: The comparisons were performed in a scenario with 50 QTLs, heritability h 2= 0.8, cofactors selected by the Method 1, and 10 backcross nested association mapping (BC-NAM) subpopulations which were randomly selected from a total of 50 BC-NAM subpopulations. The empirical type I error α ∗ was calculated based the mapping results from JICIM model and the segregating markers and QTLs within the mapping population. For details see Methods. (PDF 16.1 kb) [file 12870_2016_707_MOESM22_ESM.pdf]

Table S2 Comparisions of the power of QTL detection 1-β^* among the joint inclusive composite interval mapping (JICIM) model, joint compositve interval mapping (JCIM) model 1 and 2 in a scenario with 50 QTLs, heritability $h^2 = 0.8$, cofactors selected by the Method 1, and 10 backcross nested association mapping (BC-NAM) subpopulations which were randomly selected from a total of 50 BC-NAM subpopulations, where the empirical type I error α^* was calculated based the mapping results from JICIM model and the segregating markers and QTLs within the mapping population. For details see Materials and Methods.

Replicate	JICIM				Power 1-β^*	
	Detected QTLs	True QTLs	Power 1-β^*	α^*	JCIM 1	JCIM 2
1	76	5	0.128	0.0100	0.225	0.250
2	69	2	0.053	0.0095	0.211	0.237
3	59	3	0.075	0.0079	0.150	0.200
4	59	4	0.100	0.0078	0.075	0.225
5	69	1	0.029	0.0096	0.429	0.514
6	50	1	0.007	0.0244	0.293	0.268
7	68	1	0.026	0.0095	0.205	0.282
8	58	1	0.026	0.0080	0.205	0.231
9	62	2	0.051	0.0085	0.205	0.205
10	73	1	0.027	0.0102	0.189	0.243
Average			0.052		0.219	0.266
